# The impact of childhood maltreatment, HIV status, and their interaction on mental health outcomes and markers of systemic inflammation in women

**DOI:** 10.1186/s13293-025-00704-9

**Published:** 2025-03-28

**Authors:** Amanda Arnold, Heqiong Wang, C. Christina Mehta, Paula-Dene C. Nesbeth, Brahmchetna Bedi, Caitlin Kirkpatrick, Caitlin A. Moran, Abigial Powers, Alicia K. Smith, Kimbi Hagen, M. Neale Weitzmann, Ighovwerha Ofotokun, Cecile D. Lahiri, Jessica A. Alvarez, Arshed A. Quyyumi, Gretchen N. Neigh, Vasiliki Michopoulos

**Affiliations:** 1 Department of Psychiatry and Behavioral Sciences, Emory University School of Medicine, Atlanta, GA USA; 2 Division of Infectious Diseases, Emory University School of Medicine, Atlanta, GA USA; 3 Nutrition and Health Sciences Doctoral Program, Laney Graduate School, Emory University, Atlanta, GA USA; 4 Division of Endocrinology, Metabolism, and Lipids, Department of Medicine, Emory University School of Medicine, Atlanta, GA USA; 5 Department of Gynecology and Obstetrics, Emory University School of Medicine, Atlanta, GA USA; 6 Department of Behavioral, Social, and Health Education Sciences, Rollins School of Public Health, Emory University, Atlanta, GA USA; 7 Atlanta Department of Veterans Affairs Medical Center, Decatur, GA USA; 8 Division of Cardiology, Emory University School of Medicine, Atlanta, GA USA; 9 Department of Anatomy and Neurobiology, Virginia Commonwealth University, Richmond, VA USA; 10 Department of Psychiatry and Behavioral Sciences, School of Medicine, Emory University, Emory University, Atlanta, GA 30322 USA

**Keywords:** Childhood maltreatment, Human immunodeficiency virus, Posttraumatic stress disorder, Depression, Women, Inflammation, Lipopolysaccharide

## Abstract

**Background:**

Childhood maltreatment and HIV are both associated with a greater risk for adverse mental health, including posttraumatic stress disorder (PTSD), depression, and increased systemic inflammation. However, it remains unknown whether childhood maltreatment and HIV interact to exacerbate PTSD, depression, and inflammation in a manner that may further increase the risk of adverse health outcomes in people living with HIV. This study investigated the interaction between childhood maltreatment and HIV status on PTSD and depression symptom severity, and on peripheral concentrations of lipopolysaccharide (LPS) and high sensitivity C-reactive protein (hsCRP) in women. We hypothesized that women living with HIV (WLWH) who report high levels of childhood maltreatment exposure would show the greatest PTSD and depressive symptoms, as well as the highest concentrations of LPS and hsCRP.

**Methods:**

We conducted a cross-sectional study of 116 women (73 WLWH and 43 women without HIV). Participants completed interviews to measure trauma exposure, including childhood maltreatment, and PTSD and depression symptoms. They also provided blood samples that were analyzed for LPS and hsCRP concentrations.

**Results:**

Both women living with and without HIV reported high rates of trauma exposure and showed no statistically significant differences in overall rates of childhood maltreatment. Moderate to severe childhood maltreatment was associated with higher PTSD symptom severity (*p* =.005), greater depression severity (*p* =.005), and elevated plasma LPS concentrations (*p* =.045), regardless of HIV status. There were no effects of childhood maltreatment on hsCRP concentrations. There were no detectable significant effects of HIV status, or interactions between HIV status and childhood maltreatment, on PTSD and depression symptoms, or LPS and hsCRP concentrations (all p’s > 0.05).

**Conclusions:**

Our findings highlight the impact of childhood maltreatment on depression and PTSD symptoms and LPS concentrations in women. These results underscore the importance of trauma-informed health care in addressing childhood maltreatment to potentially improve both mental and physical health outcomes of adult women.

## Highlights

Women with higher levels of childhood maltreatment show greater PTSD and depression symptoms.

Greater childhood maltreatment was associated with elevated circulating lipopolysaccharide (LPS) levels.

Both women living with and without HIV showed markedly elevated C-reactive protein.

Results underscore the importance of considering both psychological and physiological consequences of childhood trauma in women’s health.

## Abstract (plain language summary)

Women living with HIV often face many challenges, including experiences of trauma and mental health issues. Our study looked at how childhood trauma and HIV status affect mental health and inflammation in women. We studied two groups of women: those living with HIV and those without HIV. Both groups included women who had experienced different levels of childhood trauma. We measured symptoms of depression and posttraumatic stress disorder (PTSD), as well as markers of inflammation in their blood.

We found that women who experienced more severe childhood trauma had more depression and PTSD symptoms, regardless of whether they had HIV or not. We also found higher levels of a gut-derived inflammatory marker in women who experienced more childhood trauma. Interestingly, most women with HIV in our study had well-controlled infection and HIV status itself didn’t make as much difference as we expected. This might be because both groups of women faced similar challenges, including limited access to resources and other health problems that can cause inflammation.

Our findings suggest that healthcare providers should pay attention to childhood trauma when treating women, whether they have HIV or not. This is particularly important for women from underserved communities who may face multiple health and socioeconomic challenges.

## Introduction

The HIV epidemic continues to be a significant global health challenge, with approximately 39.9 million people living with HIV (PLWH) worldwide as of 2023 [1]. The landscape of HIV has dramatically changed over the past few decades, particularly in terms of its psychological impact. HIV diagnosis is considered a Criterion A traumatic event according to the Diagnostic and Statistical Manual of Mental Disorders (DSM), due to its life-threatening nature [2]. However, advancements in antiretroviral therapy (ART) have transformed HIV from a fatal diagnosis to a manageable chronic condition, allowing PLWH to live longer lives [3]. Despite these advancements in treatment of HIV, PLWH still face numerous health challenges that can impact their well-being [4], including posttraumatic stress disorder (PTSD) [5], depression [6], and cardiometabolic disease [7]. The shift in HIV’s status from an acute, life-threatening illness to a chronic condition necessitates a broader examination of how other various social determinants of health may confer risk for adverse health outcomes to this population, beyond the HIV diagnosis itself. These social determinants, including socioeconomic status, education, race, and trauma exposure, are positioned to play a crucial role in shaping the experiences and health outcomes of PLWH, extending far beyond the direct effects of living with the virus.

Exposure to childhood maltreatment has emerged as a critical factor for conferring risk for adverse mental and physical health outcomes across the lifespan. Exposure to abuse, neglect, or household dysfunction during childhood is associated with increased risk for a wide range of mental health disorders, including PTSD, depression, and anxiety [8]. Moreover, childhood maltreatment confers risk for adverse physical health outcomes later in life, including cardiovascular disease, gastrointestinal disorders, type 2 diabetes, obesity, autoimmune disorders (such as rheumatoid arthritis and lupus), and cancer [9, 10, 11, 12, 13, 14]. Importantly, these adverse mental and physical health conditions in survivors of childhood maltreatment are often comorbid [15, 16], suggesting common underlying biological mechanisms. One biological mechanism by which childhood maltreatment impacts mental and physical health is through immune dysregulation, a mechanism that is also implicated in the high burden of disease in PLWH [13].

Childhood maltreatment is associated with elevated concentrations of inflammatory markers in adulthood such as C-reactive protein (CRP) and pro-inflammatory cytokines, including interleukin-6 (IL-6) and tumor necrosis factor-alpha (TNF-α) [13]. This low-grade inflammation induced by childhood maltreatment results from long-lasting alterations in hypothalamic-pituitary-adrenal (HPA) axis function that can result in glucocorticoid resistance [17, 18]. These long-lasting effects of childhood trauma in adulthood are linked to epigenetic changes in genes involved in stress response and immune function [18]. In addition, chronic stressors are associated with increased intestinal permeability and elevated circulating concentrations of bacterial endotoxins like lipopolysaccharide (LPS) [19, 20], which directly trigger systemic inflammation, thereby contributing to various health risks [21]. Indeed, childhood maltreatment is associated with increased incidence of gastrointestinal disorders, like irritable bowel syndrome (IBS) that are associated with increased circulating LPS [14, 22]. These biological changes contribute to the increased risk of cardiovascular disease, metabolic disorders, and other chronic health conditions observed in individuals with a history of childhood maltreatment [8].

HIV infection itself is also associated with chronic systemic inflammation, characterized by elevated levels of inflammatory markers, including CRP, and increased microbial translocation leading to higher circulating LPS concentrations [23, 24, 25]. This chronic inflammation contributes to HIV disease progression and comorbidities [23, 24, 25]. Given that both HIV and childhood maltreatment independently contribute to inflammatory processes, it becomes crucial to examine whether their interaction may lead to exacerbated inflammation in PLWH. Understanding this potential synergistic effect could have significant implications for disease management and treatment strategies in individuals living with HIV who have a history of childhood maltreatment.

The current study aimed to investigate the effects of HIV status, childhood maltreatment, and their interaction on PTSD symptom severity, depression, and markers of immune activation (LPS) and inflammation (hsCRP) in a sample of women living with HIV (WLWH) and a comparison group of sociodemographically similar women without HIV. We focused exclusively on women because they are disproportionately impacted by both HIV and childhood maltreatment, facing unique vulnerabilities and health challenges that warrant specific attention [1, 26]. We hypothesized that WLWH with a history of childhood maltreatment would demonstrate greater PTSD and depression symptom severity, along with elevated concentrations of hsCRP and LPS.

## Methods

### Participants

Participants were women recruited from 2018 to 2023 as part of the Bone, Brain, and Heart (BBH) Study, a substudy of the Atlanta site of the Multicenter AIDS Cohort/Women’s Interagency HIV Study Combined Cohort Study (MWCCS). Inclusion criteria included identifying as a cisgender female aged ≥ 30 years living either with or without HIV, completion of trauma interview, and able to provide informed consent. For WLWH, they must have had an entry plasma HIV-1 RNA of < 50 copies/mL, were currently on ART with ≥ 2 years of ART, and had creatinine clearance (CrCl) ≥ 50 mL/min estimated by the Cockcroft-Gault Eq. [27]. Exclusion criteria have been published previously [27]. Medical data regarding HIV status at time of enrollment in the current study were collected via the MWCCS. Postmenopausal status was assessed through self-report of menstrual patterns and classified via the Stages of Reproductive Aging Workshop + 10 (STRAW + 10) criteria as premenopausal, early perimenopause, late perimenopause, or postmenopausal [28]. All study procedures were approved by the Emory Institutional Review Board and the Grady Hospital Research Oversight Committee.

### Interview measures

Participants completed an interview conducted by a clinician trained to administer the study’s trauma exposure and psychological assessment instruments. Participants were compensated $50 for completing these assessments, which in total lasted up to three hours in duration. Demographic information was collected using a locally developed demographics form to assess for age, self-identified race and ethnicity, education, and annual household income [29].

Lifetime adult trauma exposure history was assessed using the Traumatic Events Inventory (TEI) [30, 31, 32]. The Childhood Trauma Questionnaire (CTQ) was used to assess childhood exposure to emotional, physical, and sexual abuse, as well as emotional and physical neglect [33]. Per standard procedure [33], individual item responses on the CTQ were summed and a group variable was created for reported rates of none-to-low (referred to in this paper as “low”) and moderate-to-severe (referred to in this paper as “high”) emotional, physical, and sexual maltreatment.

The Clinician-Administered PTSD Scale for DSM-5 (CAPS-5) was used to assess current PTSD symptoms and diagnosis based on DSM-5 criteria [34]. The CAPS is an interviewer-administered psychometrically valid and standardized diagnostic instrument that has been used previously by our group and has high interrater reliability (IRR) for the diagnosis of PTSD in similar samples (κ = 0.83; [35]). The CAPS-5 yields both a categorical diagnosis for and a dimensional score for total PTSD symptom severity. Lifetime diagnosis of PTSD and current depression diagnosis were assessed by the Mini International Neuropsychiatric Interview (MINI) [36]. The MINI was also used to assess current and lifetime psychiatric conditions including PTSD, depression, mania, hypomania, alcohol dependence/abuse, substance dependence/abuse, and schizophrenia. Depression symptom severity was assessed via the Center of Epidemiological Studies-Depression Scale (CES-D) [37].

### Blood sampling and biomarker assays

Venous blood samples were collected within two weeks of the clinical interview date in serum separation tubes (SST) and sodium citrate cell preparation tubes (CPT) by trained staff using standard phlebotomy techniques. Samples were centrifuged at 4 degrees Celsius (C) and the plasma or serum were aliquoted and frozen at -80 degrees C until the time of assay. High-sensitivity C-reactive protein (hsCRP) was measured in duplicate serum samples using a commercial ELISA (Eagle Biosciences, Amherst, NH). The assay uses a microtiter plate-immobilized monoclonal antibody to human CRP, with absorbance measured via colorimetric plate reader (Molecular Devices, San Jose, CA). The inter-assay coefficient of variation was 6%. Results were reported as mg/L, with values < 0.1 mg/L and > 10 mg/L recorded as such. Plasma lipopolysaccharide (LPS) concentrations were measured in duplicate using ELISA (MyBioSource, San Diego, CA; catalog #MBS266722) with a detection range of 15.6–1000 ng/mL. The intra-assay coefficient of variation was 17.1%. Results were reported in ng/mL.

### Statistical analyses

Demographic characteristics and trauma exposure were summarized using means and standard deviations for continuous variables, and frequencies and percentages for categorical variables. Differences between WLWH and women without HIV were assessed using independent t-tests for continuous variables and chi-square tests for categorical variables. To examine the effects of HIV status, childhood maltreatment, and their interaction on our outcomes of interest (PTSD symptoms, depression symptoms, LPS, and hsCRP concentrations), we conducted a series of two-way analyses of variance (ANOVAs). Assumptions of ANOVA were tested and met. All variables were examined for normality through multiple approaches including assessment of skewness and kurtosis, visual inspection of distributions, and Shapiro-Wilk tests. HIV status (positive vs. negative) and childhood maltreatment (none-low vs. moderate-severe) were entered as fixed factors, with each outcome measure as the dependent variable in separate models. For PTSD and depression symptoms, CAPS-5 total scores and CES-D scores were used as dependent variables for ANOVAs, respectively. For inflammatory markers, plasma LPS (ng/mL) and serum hsCRP (mg/L) concentrations were used as dependent variables for ANOVAs. To examine potential effects of adult trauma exposure on our outcomes, we conducted follow-up adjusted analyses that included adult trauma exposure as measured by the TEI as a covariate in our models. Main effects of HIV status and childhood maltreatment, as well as their interaction, were examined for each outcome. All statistical analyses were performed using SPSS (version 29.0.2.0). The significance level for all tests was set at α = 0.05, and all tests were two-tailed. Post-hoc tests with Tukey correction were conducted to assess significant interactions. Effect sizes were calculated using partial eta-squared (η_p_^2^).

## Results

### Sociodemographic characteristics

As shown in Table 1, our study sample was comprised of 116 participants: 73 WLWH and 43 women without HIV. The majority (89.7%) identified as Black, with no significant differences in racial distribution between HIV status groups (*p* =.631). The average age was similar between groups (WLWH: 50.3 years, SD = 8.55; women without HIV: 48.3 years, SD = 10.53; *p* =.266). BMI was also similar between groups (WLWH: 35.1 kg/m^2^, SD = 9.66; women without HIV: 34.1 kg/m^2^, SD = 7.67; *p* =.54. WLWH were significantly more likely to receive disability support (61.1% vs. 36.6%, *p* =.012) and to be postmenopausal (68.5% vs. 45.2%, *p* =.004) compared to women without HIV. No significant differences between groups were found in education, income, rates of psychiatric hospitalization, suicide attempts, or arrest history (Table 1). Among WLWH, 88.4% had HIV-1 RNA < 50 copies/mL (Table 1).

**Table 1:** Demographic characteristics

Characteristic	Full sample		Women without HIV		Women living with HIV		
	*n*	%	*n*	%	*n*	%	*p*-value
Race/Ethnicity (*N* = 116)							0.631
Black	104	89.7	38	88.4	66	90.4	
White	8	6.9	3	7	5	6.8	
Hispanic	1	0.9	1	2.3	0	0	
Other	3	2.6	1	2.3	2	2.7	
Highest level of education (*N* = 116)							0.324
Less than high school degree	34	29.3	15	34.9	19	26	
Graduated high school or GED	34	29.3	14	32.6	20	27.4	
Some college, college degree or graduate degree	48	41.4	14	32.6	34	46.6	
Disability Support (*N* = 113)							0.012*
No	54	47.8	26	63.4	28	38.9	
Yes	59	52.2	15	36.6	44	61.1	
Annual Income (*N* = 111)							0.163
$0 - $12,000	63	56.8	24	58.5	39	55.7	
$12,001– $24,000	23	20.7	5	12.2	18	25.7	
>$24,001	25	22.5	12	29.3	13	18.6	
Psychiatric hospitalization (*N* = 114)							0.102
No	75	65.8	23	56.1	52	71.2	
Yes	39	34.2	18	43.9	21	28.8	
Suicide Attempt (*N* = 113)							0.072
No	77	68.1	23	57.5	54	74	
Yes	36	31.9	17	42.5	19	26	
Ever been arrested (*N* = 114)							0.512
No	32	28.1	10	24.4	22	30.1	
Yes	82	71.9	31	75.6	51	69.9	
Menopause status (*N* = 115)							0.004*
Premenopausal	37	32.2	21	50	16	21.9	
Early perimenopause	6	5.2	0	0	6	8.2	
Late perimenopause	3	2.6	2	4.8	1	1.4	
Post menopausal	69	60	19	45.2	50	68.5	
HIV viral load (RNA copies/ml) (*N* = 69)							
Undetectable < 50					61	88.4	
Detected					7	10.1	
>=1000					1	1.4	

Note: * *p* <.05. HIV: Human Immunodeficiency Virus. GED: General Educational Development. RNA: Ribonucleic Acid

### Trauma exposure characteristics

As shown in Table 2, both groups reported trauma exposure, with some notable differences. WLWH reported significantly higher rates of experiencing a life-threatening illness (54.8% vs. 23.8%, *p* =.001). Conversely, women without HIV reported higher rates of being attacked with a weapon by a romantic partner (46.5% vs. 26.0%, *p* =.024) and witnessing a family member or friend attacked without a weapon (58.1% vs. 34.2%, *p* =.036). No significant differences between WLWH and women without HIV were found in other types of trauma exposure, as well as total adult trauma exposure (p’s > 0.05). There were also no significant differences in childhood maltreatment as measured by the CTQ between WLWH and women without HIV. The average CTQ scores were similar between groups (WLWH: 45.38, SD = 19.29; women without HIV: 46.27, SD = 18.29; *p* =.806), with 56% of participants reporting moderate to severe childhood maltreatment. However, participants with moderate to severe childhood maltreatment reported significantly higher levels of adult trauma compared to those with none-low childhood maltreatment (t(113) = -7.86, *p* <.001).

**Table 2:** – Trauma exposure

Characteristic	Full sample		Women without HIV		Women living with HIV		
	*n*	%	*n*	%	*n*	%	*p*-value
Natural disaster (*N* = 116)	45	38.8	21	48.8	24	32.9	0.088
Serious accident or injury (*N* = 116)	61	52.6	22	51.2	39	53.4	0.814
Life threatening illness (*N* = 115)	50	43.5	10	23.8	40	54.8	0.001*
Military combat in a war zone (*N* = 115)	3	2.6	1	2.4	2	2.7	0.907
Attacked with weapon by romantic partner (*N* = 116)	39	33.6	20	46.5	19	26	0.024*
Attacked with weapon by non-romantic partner (*N* = 116)	43	37.1	15	34.9	28	38.4	0.708
Witnessed family member or friend attacked with weapon (*N* = 116)	40	34.5	14	32.6	26	35.6	0.738
Witnessed non-family member or friend attacked with weapon (*N* = 116)	41	35.3	14	32.6	27	37	0.643
Attacked without a weapon by romantic partner (*N* = 116)	71	61.2	29	67.4	42	57.5	0.290
Attacked without a weapon by non-romantic partner (*N* = 116)	39	33.6	16	37.2	23	31.5	0.530
Witnessed family member or friend attacked without a weapon (*N* = 116)	50	43.1	25	58.1	25	34.2	0.036*
Witnessed violence between parents (*N* = 116)	42	36.2	16	37.2	26	35.6	0.738
Beaten as a child (*N* = 116)	39	33.6	17	39.5	22	30.1	0.301
Sexual contact before age 13 (*N* = 116)	46	39.7	16	37.2	40	41.1	0.679
Forced sexual contact between ages 14–17 (*N* = 116)	26	22.4	12	27.9	14	19.2	0.276
Forced sexual contact after age 17 (*N* = 116)	48	41.4	16	37.2	32	43.8	0.484
Total Adult Trauma Exposure							0.654
Childhood abuse (*N* = 116)							0.461
None - low	51	44	17	39.5	34	46.6	
Moderate - Severe	65	56	26	60.5	39	53.4	
Physical abuse							0.236
Low	15	12.9	6	14	9	12.3	
Moderate	15	12.9	2	13.3	13	17.8	
Severe	21	18.1	8	18.6	13	17.8	
Sexual abuse							0.289
Low	10	8.6	4	9.3	6	8.2	
Moderate	22	19	12	27.9	10	13.7	
Severe	34	29.3	11	25.6	23	31.5	
Emotional abuse							0.440
Low	23	19.8	6	14	17	23.3	
Moderate	10	8.6	3	7	7	9.6	
Severe	25	21.6	12	27.9	13	17.8	
Emotional neglect							0.527
Low	24	20.7	12	27.9	12	16.4	
Moderate	16	13.8	5	11.6	11	15.1	
Severe	12	10.3	4	9.3	8	11	
Physical neglect							0.210
Low	12	10.3	2	4.7	10	13.7	
Moderate	9	7.8	3	7	6	8.2	
Severe	12	10.3	7	16.3	5	6.8	

Note: *p <.05. **HIV**: Human Immunodeficiency Virus

### Effects of HIV status, childhood maltreatment and their interaction on mental health

#### Depression

**Fig. 1:**
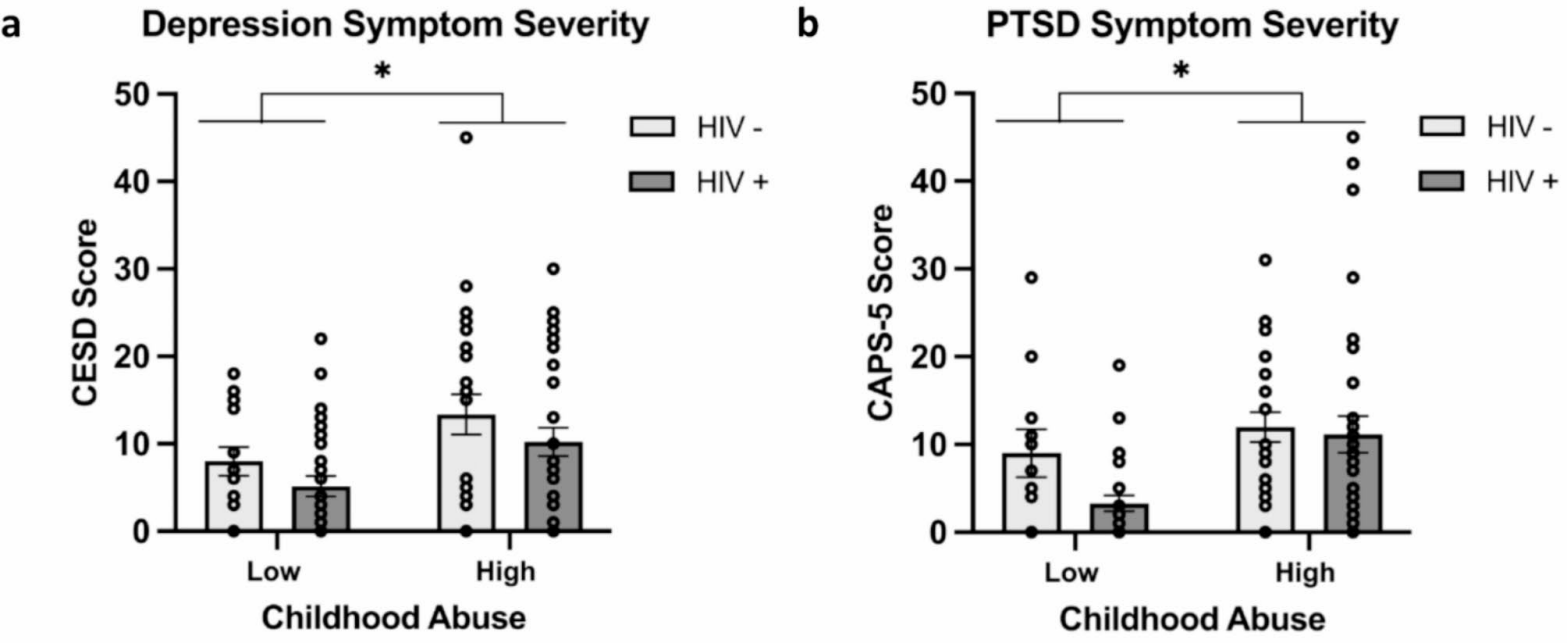
Impact of childhood maltreatment on depression and PTSD symptom severity in WLWH and women without HIV. (**a**) Depression symptom severity as measured by Center of Epidemiological Studies-Depression Scale (CESD) scores (*N* = 101). (**b**) PTSD symptom severity as measured by Clinician-Administered PTSD Scale for DSM-5 (CAPS-5) scores (*N* = 97). Both graphs show symptom severity for low (none-low) and high (moderate to severe) levels of childhood maltreatment in women without HIV (white bars) and WLWH (gray bars) women. Asterisks (*) indicate significant main effects of childhood maltreatment on symptom severity (*p* <.05). Individual data points are represented by circles. Error bars represent standard error of the mean

There were no significant differences between current or lifetime diagnosis of depression between HIV status groups (p’s > 0.05; Table 3). When examining the effects of childhood maltreatment severity and HIV status on depression symptoms we found a significant main effect of childhood maltreatment on depression symptoms (F(1, 97) = 8.11, *p* =.005, η_p_^2^ = 0.077), with participants who experienced moderate to severe maltreatment reporting higher levels of depressive symptoms than those with none-low maltreatment (Fig. 1a). We did not detect a significant main effect of HIV status on depression symptoms (*p* =.139) and no significant interaction between HIV status and childhood maltreatment (*p* =.986). In our adjusted model co-varying for adulthood trauma, the effect of childhood maltreatment remained significant (F(1, 95) = 9.04, *p* =.003, η_p_^2^ = 0.087) and the effects of HIV status remained not significant (*p* =.149), with no significant interaction between HIV status and childhood maltreatment (*p* =.835).

**Table 3:** Mental health and substance use

Characteristic	Full sample		Women without HIV		Women living with HIV		
	*n*	%	*n*	%	*n*	%	*p*-value
PTSD (*N* = 111)							0.107
Current (meets CAPS criteria)	13	11.7	9	20.9	4	5.9	
Past (lifetime)	37	33.3	13	30.2	24	35.3	
Depression (*N* = 111)							0.347
Current	20	18	9	21.4	11	15.9	
Past (lifetime)	31	27.9	14	33.3	17	24.6	
Bipolar Spectrum Disorders							
Mania (*N* = 112)							0.235
Current	3	2.7	1	2.4	2	2.9	
Past (lifetime)	4	3.6	3	7.1	1	1.4	
Hypomania- lifetime (*N* = 111)	4	3.6	2	4.8	2	2.9	0.581
Substance Use Disorders							
Alcohol Dependence(*N* = 112)							0.687
Current	7	6.3	2	4.7	5	7.2	
Past (lifetime)	27	24.1	12	27.9	15	21.7	
Alcohol abuse (*N* = 112)							0.674
Current	7	6.3	2	4.7	5	7.2	
Past (lifetime)	16	14.3	5	11.6	11	15.9	
Substance dependence (*N* = 112)							0.127
Current	13	11.6	3	7	10	14.5	
Past (lifetime)	32	28.6	9	20.9	23	33.3	
Substance abuse (*N* = 112)							0.517
Current	8	7.1	2	4.7	6	8.7	
Past (lifetime)	27	24.1	11	25.6	16	23.2	
Schizophrenia (*N* = 110)							0.147
Current	5	4.5	3	7.3	2	2.9	
Past (lifetime)	4	3.6	3	7.3	1	1.4	

Note: Assessed by the MINI. Current diagnoses reflect symptoms present within the past month. Past (lifetime) diagnoses reflect symptoms experienced at any point in the participant’s life. HIV: Human Immunodeficiency Virus. PTSD: Post-traumatic Stress Disorder. MINI: Mini International Neuropsychiatric Interview. CAPS: Clinician-Administered PTSD Scale

#### PTSD

There were no significant differences between current diagnosis of PTSD assessed by the Clinician-Administered PTSD Scale (CAPS) or lifetime diagnosis of PTSD assessed by the Mini International Neuropsychiatric Interview (MINI) between HIV status groups (p’s > 0.05; Table 3). When examining the effects of childhood maltreatment and HIV status on PTSD symptoms we found a significant main effect of childhood maltreatment (F(1, 93) = 8.17, *p* =.005, η_p_^2^ = 0.081), with participants who experienced moderate to severe childhood maltreatment reporting higher levels of PTSD symptoms than those with none to low maltreatment (Fig. 1b). We did not detect a significant main effect of HIV status (*p* =.140) and no significant interaction between HIV status and childhood maltreatment on PTSD symptoms (*p* =.281). When adjusting for adult trauma exposure in our analyses, adult trauma emerged as a significant covariate (F(1, 91) = 5.85, *p* =.018, η_p_^2^ = 0.06), while the effect of childhood maltreatment became non-significant (*p* =.262). The effect of HIV status on PTSD symptoms remained non-significant (*p* =.072), as did the interaction between HIV status and childhood maltreatment (*p* =.439).

#### Other mental health and substance use conditions

We also assessed a range of conditions using the MINI (Table 3), including psychiatric disorders (mania, hypomania, and schizophrenia) and substance use disorders (alcohol and drug dependence/abuse). Both current and lifetime diagnoses were evaluated where applicable. There were no significant differences between WLWH and women without HIV for any of these conditions in our sample (all p’s > 0.05).

### Effects of HIV status, childhood maltreatment and their interaction on immune activation and inflammatory markers

#### LPS

**Fig. 2:**
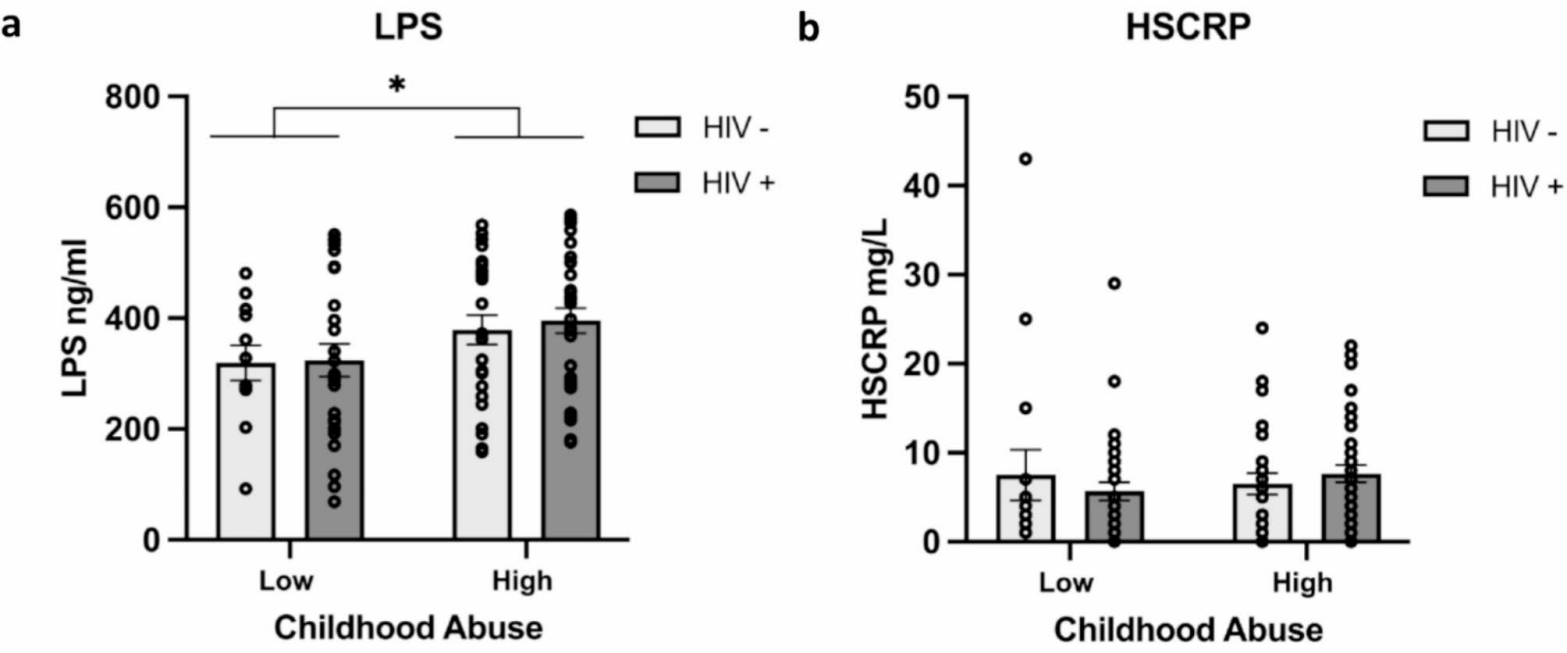
Impact of childhood maltreatment on inflammatory markers in WLWH and women without HIV. (**a**) Plasma lipopolysaccharide (LPS) concentrations (*N* = 96). (**b**) High-sensitivity C-reactive protein (hsCRP) concentrations (*N* = 113). Both graphs show inflammatory marker concentrations for low (none-low) and high (moderate to severe) levels of childhood maltreatment in women without HIV (white bars) and WLWH (gray bars). Asterisk (*) in panel (**a**) indicates a significant main effect of childhood maltreatment on LPS concentrations (*p* <.05). No significant differences were observed for hsCRP concentrations. Individual data points are represented by circles. Error bars represent standard error of the mean. LPS is measured in ng/ml, and hsCRP is measured in mg/L

A significant main effect of childhood maltreatment was found on plasma LPS concentrations (F(1, 92) = 4.13, *p* =.045, η_p_^2^ = 0.043 ), with individuals who experienced moderate to severe childhood maltreatment showing higher concentrations of plasma LPS. We did not detect a significant effect of HIV status on LPS concentrations (*p* =.846), and there was no significant interaction between HIV status and childhood maltreatment (*p* =.959) on LPS concentrations (Fig. 2a). In our adjusted model co-varying for adulthood trauma, the effect of childhood maltreatment on plasma LPS concentrations remained significant (F(1, 90) = 4.19, *p* =.044, η_p_^2^ = 0.044 ), while HIV status effects remained not significant (*p* =.907), with no significant interaction (*p* =.943).

#### hsCRP

We found no significant main effects of HIV status (*p* =.845) or childhood maltreatment (*p* =.729), and no significant interaction between these factors on hsCRP concentrations (*p* =.315) (Fig. 2b). In our adjusted model co-varying for adulthood trauma, the effects of childhood maltreatment (*p* =.238), HIV status (*p* =.430) and their interaction (*p* =.253) on hsCRP concentrations remained not significant.

## Discussion

In the current study, we assessed the effects of HIV status, childhood maltreatment, and their interaction on mental health outcomes and inflammatory markers in a sample of WLWH and women without HIV. Our results indicate that moderate to severe childhood maltreatment is associated with higher PTSD symptom severity, greater depression severity, and elevated plasma LPS concentrations. We did not observe any influence of HIV status nor an interaction between HIV status and childhood maltreatment on the outcomes we assessed. Our findings suggest that childhood maltreatment plays a crucial role in influencing depression and PTSD symptoms and LPS levels in adulthood, and highlight the need for trauma-informed care in treatment and prevention settings for women facing multiple socioeconomic challenges and health disparities. It is important to note that our findings should be interpreted within the context of our specific sample characteristics, which include high rates of childhood maltreatment exposure and similar socioeconomic challenges in WLWH and women without HIV. The average rates of childhood maltreatment in both WLWH (45.38) and women without HIV (46.27) exceed those typically found in community populations (31.7) [38] and highlight the substantial trauma burden faced by women in this study. Our participants also experienced significant socioeconomic challenges, with 56.8% reporting annual incomes of $12,000 or less, 29.3% having less than a high school education, and 52.2% receiving disability support. In addition, 71.9% of participants reported a history of arrest, further reflecting the systemic disadvantages experienced by this predominantly Black (89.7%) female sample.

Our first main finding was that moderate to severe childhood maltreatment was associated with greater PTSD and depression symptoms. These findings align with extensive literature documenting the long-term consequences of childhood trauma on mental health [9, 39]. We also found that the effect of childhood maltreatment on PTSD symptoms was mediated by adult trauma exposure, which was significantly greater in individuals who had experienced moderate to severe childhood trauma in our sample. This pattern aligns with literature on re-victimization and risk for PTSD, where individuals exposed to childhood maltreatment are at increased risk for subsequent trauma exposure and PTSD in adulthood [40, 41]. This finding highlights the importance of assessing lifetime trauma burden rather than focusing solely on early life adversity, as the cumulative and interactive effects of trauma across the lifespan are particularly relevant for PTSD symptoms. Our results differ from recent work that reported an association between HIV and PTSD symptom severity in women with low levels of childhood maltreatment [42]. This could be due to participant differences between the two studies. Specifically, WLWH participants described in the original report had higher levels of education and income compared to participants without HIV. In contrast, the study sample of WLWH and women without HIV included in the current study were more economically homogeneous between groups, with over half (56.8%) of all participants reporting an annual income of $12,000 or less. This disparity in socioeconomic distribution between the two study samples may contribute to the differences in our findings such that HIV interaction effects may be masked due to limited access to resources seen in our cohort.

Results also indicated that moderate to severe childhood maltreatment was associated with greater plasma LPS concentrations. Childhood maltreatment can act as a chronic stressor to impact intestinal barrier function through HPA axis dysregulation and altered sympathetic nervous system activity, which affect both tight junction proteins and gut microbiome composition [43, 44]. These changes can lead to increased intestinal permeability and subsequent microbial translocation, as evidenced by elevated LPS concentrations [24, 43, 44]. The association between childhood maltreatment and increased plasma LPS concentrations is particularly noteworthy as it suggests a potential link between early life trauma and chronic immune activation. Elevated LPS concentrations indicate increased microbial translocation, which can contribute to systemic inflammation and associated health risks. While previous research has linked childhood maltreatment to gut health [13, 15, 45], our finding of increased LPS concentrations provides new insights into the potential gut-brain axis involvement in the long-term effects of childhood trauma. This finding underscores the importance of considering childhood trauma not only in the context of mental health but also as a potential factor influencing physiological processes that contribute to disease progression through alterations in gut permeability.

Interestingly, we did not detect significant main effects of HIV status on depression severity nor inflammatory markers assessed. These null results are in contrast with previous research suggesting higher rates of mental health burden and greater inflammation among PLWH compared to those without HIV [23, 24, 46]. Our findings, however, align with more recent studies suggesting that improvements in HIV treatment and potential reductions in stigma might be narrowing the gap in mental health disparities between PLWH and the general population [47]. The lack of HIV effects in our study must be interpreted in the context of our highly virally-suppressed sample, where 88.4% of WLWH had HIV-1 RNA < 50 copies/mL. This high rate of viral suppression reflects current standards of HIV care, where antiretroviral therapy effectively manages HIV infection and may minimize its impact on inflammatory and psychological outcomes. Our findings therefore should not be generalized to individuals with uncontrolled HIV infection or those not receiving optimal treatment. Rather, our results provide insight into the relative contributions of childhood trauma and well-managed HIV in the modern treatment era.

The lack of significant differences between groups based on HIV status might also be partially explained by the lack of differences in body mass index (BMIs) between HIV status groups (average BMI of 35 in WLWH and 34 in women without HIV). Obesity is independently associated with elevated inflammatory markers including hsCRP and LPS [48, 49] and increased risk for depression and other mental health conditions [50]. While previous research has shown associations between childhood maltreatment and increased risk for obesity in adulthood [51, 52], we did not find this relationship in our sample. This lack of association may be due to the overall high BMI observed across our sample regardless of trauma exposure. These consistently high BMIs across groups might have contributed to the elevated inflammatory markers we observed in our sample regardless of trauma exposure or HIV status. In addition, our comprehensive psychiatric assessment revealed substantial psychiatric comorbidity in both groups, with particularly high rates of lifetime substance use disorders (~ 25–29%) and similar patterns of psychiatric burden between WLWH and women without HIV. These similarly high BMIs and psychiatric comorbidities across both groups might have created a “ceiling effect” of inflammation and associated symptoms that could mask any additional inflammatory or psychological effects specifically attributable to HIV status and supports our observation that mental health outcomes in this population may be more strongly influenced by trauma exposure and other social determinants of health than by HIV status alone [53].

It is also important to note that concentrations of hsCRP in our study sample were markedly elevated compared to the general population [54] and were not shown to be influenced by HIV status or childhood maltreatment. This contradicts previous research linking these factors to chronic inflammation [13, 55, 56]. The lack of HIV and childhood maltreatment effects on hsCRP in our study may be due to various factors, including the chronic nature of both HIV and the potential effects of antiretroviral therapy on inflammation in WLWH [57]. In addition, study participants, regardless of HIV status, who face multiple socioeconomic challenges reported disproportionally high rates of cumulative trauma exposure over the life course. This finding has been reported in similar studies of minoritized and under resourced women in the Atlanta area [58]. This increased trauma burden, along with other social determinants of health that we did not directly measure (such as access to healthcare, neighborhood characteristics, or experiences of discrimination), may contribute to overall elevated inflammation levels across our sample, potentially masking group differences by creating a ceiling effect on hsCRP concentrations. These findings underscore the need for comprehensive assessments and interventions that address the social determinants of health in urban, low-income populations, particularly among Black women.

The current study has several limitations that should be considered. The cross-sectional nature of our data precludes causal inferences about the relationships between variables. While our sample size was sufficient to detect the observed effects of childhood maltreatment, it may have limited our ability to detect smaller effects. Larger studies with more balanced group sizes may be needed to definitively establish the presence or absence of small effects related to HIV status or potential interactions between HIV status and childhood trauma. Another limitation is that we did not directly measure current perceived stress levels. Given that chronic stress is associated with elevated inflammatory markers [59], future studies should include measures of current perceived stress to better understand how ongoing stressors may interact with early life trauma to influence health outcomes.

While our study’s focus on women provides important insights into childhood maltreatment and HIV effects in this population, it limits our ability to generalize our findings to men and to examine potential sex differences in these relationships. Previous literature suggests significant sex differences in both trauma responses and inflammation. Women generally show higher rates of PTSD following trauma exposure [60, 61] and exhibit different inflammatory profiles compared to men [62]. Some evidence suggests that women may be particularly vulnerable to stress-induced alterations in immune function [63]. In addition, while our focus on an under-resourced, predominantly minoritized sample of women is crucial for addressing health disparities, it may limit the generalizability of our findings to more affluent or demographically different populations. It is important to note, however, that this focus allows us to examine these issues in a population disproportionately affected by both HIV and trauma exposure [64, 65]. The relationships we observed between childhood trauma, mental health, and inflammation may manifest differently in populations with greater socioeconomic resources or different racial/ethnic backgrounds [66, 67]. For instance, access to mental health care, stress-buffering resources, and quality medical care could modify the impact of childhood trauma on later health outcomes [68]. In addition, our sample’s high rates of obesity and psychiatric comorbidities may reflect specific challenges faced by urban, low-income populations, potentially limiting generalizability to populations with different health profiles.

Future longitudinal research in women and men is necessary to better understand the temporal dynamics between trauma exposure, HIV status, mental health, and immunological outcomes in the context of chronic, comorbid adverse health conditions. Investigating potential mediators and moderators that may influence the effects of childhood maltreatment and HIV status on health, such as coping strategies, social support, and access to mental health care, could provide valuable insights for prevention and intervention development. In addition, incorporating measures of HIV disease progression in WLWH and examining a broader range of biological mechanisms could help fill the current gap in knowledge regarding the interactions between trauma exposure, mental health, and physical health outcomes in PLWH. These future studies could examine fear psychophysiology and glucocorticoid functioning, as both systems can be impacted by HIV [69, 70] and childhood maltreatment [9, 17, 71].

In conclusion, our results highlight the enduring impact of childhood maltreatment on PTSD and depression symptoms and LPS concentrations in a sample of under resourced, predominantly minority women facing multiple socioeconomic challenges, regardless of HIV status. However, our results must be interpreted within important contextual limitations: our WLWH participants were predominantly virally-suppressed, both women living with and without HIV had similarly high BMIs and comparable psychiatric comorbidities. Nevertheless, our findings highlight the need for integrated, trauma-informed approaches in health care and prevention that address both the psychological and immunological sequelae of early life trauma. By recognizing and addressing the trauma histories of women, particularly those from marginalized communities with limited access to resources, we can work towards more effective interventions that improve both health outcomes and overall well-being in this most affected population.

## Data Availability

The data that support the findings of this study are not openly available due to reasons of sensitivity and are available from the corresponding author upon reasonable request.
